# Supplementary material for: Stromal expression of hemopexin is associated with lymph-node metastasis in pancreatic ductal adenocarcinoma
Source: PLoS One. 2020 Jul 14;15(7):e0235904. doi: 10.1371/journal.pone.0235904 (PMC7360047; doi:10.1371/journal.pone.0235904)
Supplement: S1 Table — Fold change and significance for 9 proteins selected on the volcano plot. AGR3, anterior gradient 3, protein disulfide isomerase family member; DEF3, α-defensin 3; MYH14, myosin heavy chain 14; ABHD14B, abhydrolase domain containing 14B; FTL, ferritin light chain; TPM1, α-tropomyosin; CSRP1, cysteine- and glycine-rich protein 1. (DOCX) [file pone.0235904.s001.docx]

**S1 Table. Fold change and significance for the candidate proteins**

| Protein | -Log Student's T-test p-value N0_N1 (Significance) | Student's T-test Difference N0_N1 (Fold change) |
| --- | --- | --- |
| AGR3 | 1.472720222 | 2.06380043 |
| DEF3 | 1.494039435 | 4.086812019 |
| MYH14 | 1.360788362 | 1.735110664 |
| ABHD14B | 1.372167698 | 2.957462502 |
| Hemopexin | 3.622285512 | -2.029213905 |
| FTL | 2.440395107 | -3.904712868 |
| CSRP1 | 1.462670539 | -1.78057251 |
| TPM1 | 1.382090548 | -2.736831474 |
| Plectin | 1.342581491 | -2.188614273 |

Fold change and significance for 9 proteins selected on the volcano plot. AGR3, anterior gradient 3, protein disulfide isomerase family member; DEF3, α-defensin 3; MYH14, myosin heavy chain 14; ABHD14B , abhydrolase domain containing 14B; FTL, ferritin light chain; TPM1, α-tropomyosin; CSRP1, cysteine- and glycine-rich protein 1
